# A Knowledge-Guide Data-Driven Model with Selective Wavelet Kernel Fusion Neural Network for Gearbox Intelligent Fault Diagnosis

**DOI:** 10.3390/s25247656

**Published:** 2025-12-17

**Authors:** Nan Zhuang, Zhaogang Ren, Dongyao Yang, Xu Tian, Yingwu Wang

**Affiliations:** 1 Department of Environmental Science and Engineering, China West Normal University, Nanchong 637000, China; 2zhuangn@stu.cwnu.edu.cn (N.Z.);; 2 College of Science, Technology, Engineering and Mathematics, Royal Melbourne Institute of Technology University, Melbourne, VIC 3001, Australia

**Keywords:** wavelet convolution, modern TCN, selective fusion, deep learning, fault diagnosis

## Abstract

The gearbox is a critical component in modern industrial systems, directly determining the operational reliability of machinery. Therefore, effective fault diagnosis is essential to ensure its proper functioning. Modern diagnostic approaches often employ accelerometers to monitor vibration signals and apply data-driven techniques for fault identification, achieving considerable success. However, deep learning-based methods still face limitations due to their “black-box” nature and lack of interpretability. To address these issues, this paper proposes a knowledge-guided selective wavelet kernel fusion neural network. By integrating diagnostic domain knowledge into data-driven modeling, the proposed method enhances both the interpretability and diagnostic performance of intelligent fault diagnosis systems. First, a multi-kernel convolutional module is designed based on domain knowledge and embedded into a Modern Temporal Convolutional Network. Then, an attention-based selective wavelet kernel fusion strategy is introduced to adaptively fuse kernels according to the distribution of different datasets. Finally, the effectiveness of the proposed method is validated on two public datasets. Experimental results demonstrate that the approach not only provides prior interpretability, which overcoming the black-box limitation of deep learning, but also further improves diagnostic accuracy.

## 1. Introduction

Gearboxes are of critical importance in modern industry, with a wide range of applications across various sectors such as aerospace, wind power generation, automotive manufacturing, and mining machinery. Accordingly, the performance and reliability of these systems have become key factors that influence both the operational efficiency and safety of equipment [1]. Therefore, gearbox fault diagnosis is highly significant, as it not 36 only improves production stability but also reduces maintenance costs and extends equip-37 ment service life [2,3].

The rapid advancement of artificial intelligence (AI) technologies—such as deep learning and machine learning—coupled with the proliferation of Internet of Things (IoT) technologies like high-frequency vibration sensors and high-speed communication systems, has laid a solid technical foundation for data-driven fault diagnostics. The deployment of such sensors facilitates the acquisition of large-scale, multi-dimensional datasets, which in turn can be leveraged to power diagnostic processes. Recent progress in this area has been characterized by the development of sophisticated deep learning-based diagnostic techniques, including Convolutional Neural Networks (CNNs) [4], Long Short-Term Memory networks (LSTMs) [5], Graph Neural Networks (GNNs) [6], and Transformers [7]. These methods have enabled the creation of autonomous models capable of identifying complex fault characteristics directly from raw data, thereby improving diagnostic autonomy and diminishing the dependence on human expertise. In parallel, advances in signal processing have been integrated with intelligent modeling frameworks—for instance, the combination of envelope spectral analysis with spectral kurtosis [8]. Such methodologies typically first apply signal processing techniques to convert raw signals into more interpretable representations, which are then fed into AI models for diagnosis. Despite the notable effectiveness of these approaches, they collectively face a common challenge: the limitations inherent in the so-called “black box” nature of the models. Although these systems are capable of delivering highly accurate diagnostic outcomes, they still fall short in providing comprehensive rationales for their decisions or in elucidating the underlying physical principles that govern their operation.

Wavelet analysis is a classical method that enables comprehensive signal analysis from a time–frequency perspective. Within the domain of fault diagnosis, it facilitates joint time–frequency analysis of signals through a scalable and shiftable mother wavelet function. The high-frequency component can locate the instantaneous occurrence of transient impacts, while the low-frequency component can identify fault characteristics. In the current era of deep learning development, the integration of wavelet analysis with deep learning represents a significant undertaking. Initial approaches entailed the conversion of signals into two-dimensional data via wavelet transformation, followed by pattern recognition utilizing computer vision processing methods. Nevertheless, the efficacy of such methodologies was found to be inadequate, resulting in an inadequate level of integration. Subsequently, Li et al. [9] sought to enhance the intelligence of wavelet transforms embedded within DL by establishing a closed-loop system for backpropagation-based network parameter updates. The wavelet kernel was replaced with a convolutional kernel, and the scale factor and shift factor were designed as learnable parameters. This enabled intelligent adjustment of the wavelet transform process. Furthermore, Jiang et al. [10] designed a network integrating multiple wavelet kernels, employing four distinct kernels for comprehensive feature extraction. Even though the approach of integrating multiple wavelet kernels is fundamentally sound, it must be noted that it introduces information redundancy. The optimal scenario entails the selection of suitable wavelet kernels for fusion and embedding, based on the data distribution within the dataset, followed by feature learning.

A knowledge-guided, data-driven approach is essential to unveiling the mechanisms behind the black box and building trustworthy, highly robust intelligent diagnostic systems for industrial applications. In data science, several methods have been adopted to narrow the gap between the “black box” and the desirable “white box” paradigm—the latter referring to a data-driven system with transparent internal logic. These techniques include data augmentation, model design, and loss function design [11], among others. By applying such strategies, it becomes possible to integrate physical-world insights into data-driven models, thereby promoting a shift from opaque systems toward more interpretable and understandable ones. Among these strategies, certain model architectures stand out for their ingenuity, incorporating mathematical expressions of physical principles directly into the network structure. The application of such models has been shown to provide significant advantages, including improved interpretability, greater data efficiency, and stronger physical consistency. The primary benefit of incorporating mechanistic knowledge is the replacement of certain data-driven modules with physical model calculations. This approach renders the computational process independent of neural networks, thereby further streamlining the entire model. This optimisation renders it well-suited for edge deployment scenarios [12,13,14].

To address the aforementioned challenges, this paper proposes a knowledge-guided data-driven diagnostic approach based on mainstream accelerometer-based vibration signal monitoring. The method incorporates a Multiwavelet Kernel Convolution (MWC) module and a selective kernel fusion strategy, rooted in domain knowledge of fault diagnosis, into a Modern Temporal Convolutional Network (ModernTCN). This integration enables end-to-end feature learning and fault diagnosis directly from raw vibration signals. The main contributions of this work are summarized as follows:

1. Leveraging expertise in wavelet kernel matching within the fault diagnosis domain, a Multiwavelet Kernel Convolution (MWC) module is designed to enhance the model’s prior interpretability and improve feature extraction.
2. The MWC module is integrated into the ModernTCN to construct the backbone network for diagnosis.
3. An attention-based selective kernel fusion mechanism is developed which adaptively learns weighting coefficients and applies the Softmax function to normalize them into independent probabilities.
4. Experiments conducted on two public gearbox fault datasets validate the effectiveness of the proposed method.

## 2. Selective Wavelet Kernel Fusion Neural Network

### 2.1. Multiwavelet Kernel Convolution Module

Utilizing accelerometers for condition monitoring of gearboxes is a well-established technique. During gearbox operation, periodic resonance phenomena occur due to gear tooth meshing and rolling friction between bearing balls and inner race and outer race, which can be effectively captured by monitoring systems. When faults develop in the gearbox, they induce abnormal impulse vibrations that are readily detected by accelerometers. Traditional signal processing methods rely on manual analysis based on empirical knowledge to identify abnormal impulse components, particularly fault characteristic frequencies, for condition assessment.

In the current data-driven era, employing deep learning models to automatically process vibration data for fault detection and diagnosis has become a mature methodology. However, deep learning-based approaches suffer from the black-box limitation, making it difficult to interpret their decision-making processes. To address this, Li et al. [9] proposed a wavelet kernel convolution method which replaces conventional convolutional kernels with wavelet kernels. By adaptively learning the scale and shift factors of the wavelet kernels, this approach provides prior interpretability to the model.

**Figure 1:**
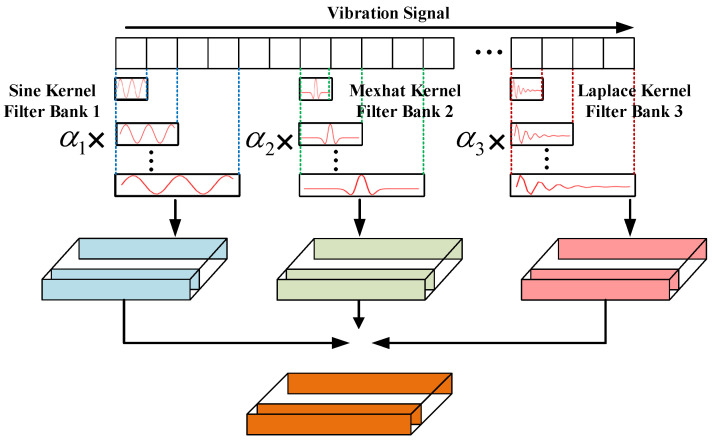
Structure of MWC module.

To further overcome the limitations of deep learning in fault diagnosis, this paper designs a multi-wavelet kernel convolution module that employs three distinct wavelet kernel functions for feature extraction: the sine wavelet for capturing periodic characteristics, the Mexican hat (Mexhat) wavelet for extracting impulse features, and the Laplace wavelet for identifying impulse decay information [10]. This configuration enables effective extraction of the three primary characteristics present in vibration signals. A schematic diagram of the MWC module is shown in Figure 1, with the fundamental formulation expressed as follows:

(1) $$f=\sum_{i}\left[\Psi_{i}*x(t)\right]$$

(2) $$\Psi_{i}=\{\Psi_{\mathrm{sine}},\Psi_{\mathrm{Mexhat}},\Psi_{\mathrm{Laplace}}\}$$

(3) $$\left\{\begin{array}{l} \Psi_{sine}=sin(t)\\ \Psi_{Mexhat}=(1-t^{2})e^{\frac{t^{2}}{2}}\\ \Psi_{Laplace}=Ae^{\frac{-\xi }{\sqrt{1-\xi^{2}}}2\pi f(t-\eta )}\times sin[2\pi f(t-\eta )] \end{array}\right.$$

where f is the output feature map of MWC module; $\Psi_{i}$ are the wavelet kernel functions; A is the normalized constant of the wavelet; ξ is the viscous damping ratio; and η is the delay parameter. In this paper, real part of the Laplace wavelet is utilized as the kernel function to process the vibration data. Although the designed MWC module replaces conventional convolution kernels with wavelet kernels, it is necessary to perform backpropagation during the deep learning training process to update and converge the scale and translation factors of the wavelets within the module. The backpropagation formulas after substituting convolution kernels with wavelet kernels are derived as follows [10]:

(4) $$\left\{\begin{array}{l} \delta_{\tau }=\frac{\partial L}{\partial \tau }=\frac{\partial L}{\partial p}\frac{\partial p}{\partial \Psi }\frac{\partial \Psi }{\partial \tau }\\ \tau =\tau -\eta \tau \end{array}\right.$$

(5) $$\left\{\begin{array}{l} \delta_{s}=\frac{\partial L}{\partial s}=\frac{\partial L}{\partial p}\frac{\partial p}{\partial \Psi }\frac{\partial \Psi }{\partial s}\\ s=s-\eta s \end{array}\right.$$

where δ is the gradient; τ and s are the translation and scale factors, respectively; L is the loss function; p is the output feature map of previous network layer; Ψ is the wavelet kernel filter; and η is the learning rate.

This feature extraction module incorporates domain knowledge of wavelet-function matching with impulse characteristics in fault diagnosis, while being implemented as a data-driven convolutional neural layer. It effectively integrates the advantages of both knowledge guidance and data-driven learning, thereby providing the model with prior interpretability.

### 2.2. ModernTCN with MWC

To incorporate the wavelet kernel function into the mainstream neural network, this paper utilizes the Modern Temporal Convolution Network (ModernTCN) [15] as the backbone network. The MWC module contains multiwavelet kernel convolution. Each wavelet kernel convolution can capture the specific fault-related information in vibration, it means that multiwavelet kernel convolution is a multibranch structure. So that this paper add an additional wavelet kernel weight to adjust the importance of each wavelet. The key is that how designs the best weight generation method to leverage the feature extraction ability of each wavelet kernel convolution. In Section 2.3, this paper will further introduce the proposed weight generation strategy.

**Figure 2:**
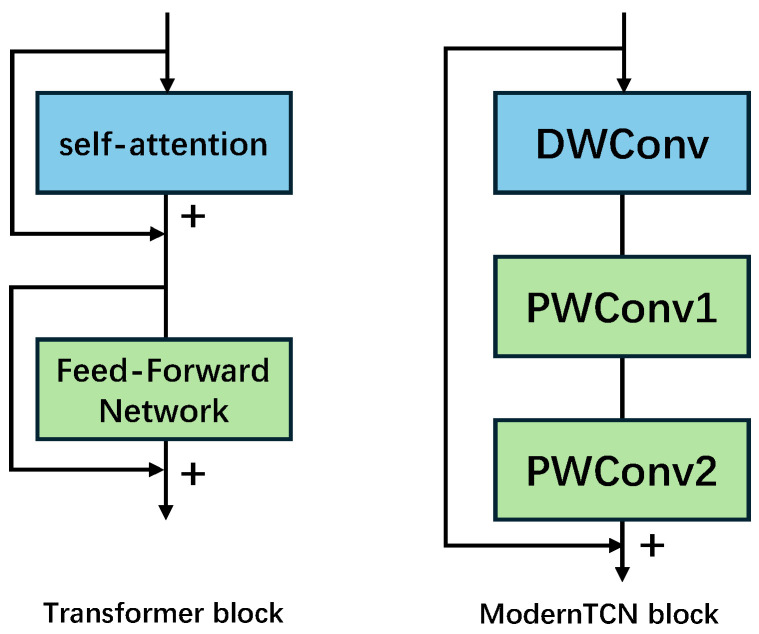
Deference between transformer block and ModernTCN block.

ModernTCN is a modified CNN which it inspired by modern self-attention mechanism-based transformer structure. Basic convolution module of standard CNN contains convolution layer, normalization layer, activation layer and pooling layer. And multiple convolution modules stack to construct the CNN-like network. Traditional CNN-like network can not efficient process the time sequence for any task. Thereby, the temporal convolution network was proposed to overcome the limitation of standard CNN by dilation convolution layer. Also self-attention mechanism-based transformer model can best face the time sequence task, but it needs the large computation source and time expenditure. Thereby, the ModernTCN modify the transformer block. The self-attention replaced by depth-wise convolution (DWConv) [16] and the Feed-Forward Network replaced by two stacked point-wise convolutions (PWConv) [17]. The difference between transformer block and ModernTCN block is shown in Figure 2. In the ModernTCN block, the DWConv to model the time relationship, the first PWConv to model channel relationship, and the second PWConv to model variable relationship.

To embed the MWC module into ModernTCN, it only needs to feed the output feature map of MWC module into first block of ModernTCN.

### 2.3. Selective Kernel Fusion

The proposed Selective Wavelet Kernel Fusion Network is designed to adaptively integrate multiple wavelet kernels based on the intrinsic characteristics of vibration signals for rotating machinery fault diagnosis. The framework comprises two main components: (1) a self-attention-based weight-generation module, (2) a selective fusion mechanism with thresholding.

The core innovation of our approach lies in the adaptive weight generation mechanism that dynamically assigns importance scores to different wavelet kernels. Given an input vibration signal, we first employ a self-attention mechanism to capture the global dependencies and salient features:

(6) $$Q=XW_{Q},K=XW_{K},V=XW_{V}$$

where $W_{Q}$, $W_{K}$, $W_{V}\in {\mathbb{R}}^{\left\{L\times d\right\}}$ are learnable projection matrices; and L denote the signal length; d represents the hidden dimension. The attention weights are computed as:

(7) $$A=softmax(QK^{T}/\sqrt{d})$$

The contextual representation is obtained through:

(8) Z=AV

Subsequently, the attention-enhanced features Z are fed into a Multi-Layer Perceptron (MLP) to generate the wavelet kernel weights:

(9) $$\begin{array}{l} w_{raw}=MLP(GAP(Z))\\ w=\sigma (w_{raw}) \end{array}$$

where σ(·) denotes the sigmoid activation function, ensuring that each weight value $\begin{array}{cc} w_{i}\in [0,1] & i=1,2,3 \end{array}$. The three weights correspond to sine, mexhat, and laplace wavelet kernels, respectively.

To achieve selective kernel fusion, we introduce a threshold mechanism that determines the participation of each wavelet kernel in the feature extraction process. For each wavelet kernel weight $w_{i}$, the fusion decision is governed by:

(10) $$\alpha_{i}=\left\{\begin{array}{ll} w_{i}, & w_{i}>\tau \\ 0, & otherwise \end{array}\right.$$

where *τ* = 0.65 is the empirically determined threshold, detailed setting is conducted in Section 3.5. This selective mechanism ensures that only the most relevant wavelet kernels contribute to the final feature representation, thereby enhancing the model’s discriminative capability while reducing redundant computations.

The fused wavelet feature representation is computed as:

(11) $$F_{fusion}=\sum_{i=1}^{3}\alpha_{i}\cdot (\Psi_{i}*X)$$

where the summation is normalized by the number of active kernels to maintain numerical stability.

### 2.4. Network Architecture and Training Process

**Figure 3:**
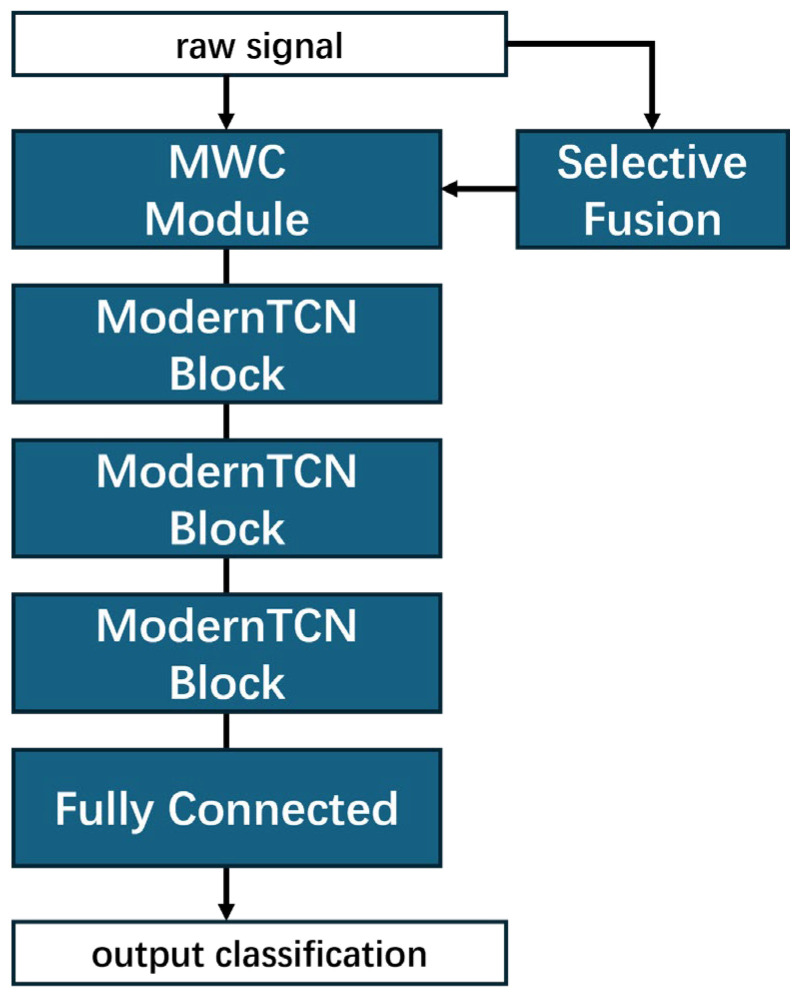
The architecture of proposed selective wavelet kernel fusion network.

Figure 3 illustrates the overall architecture of the proposed method. The first layer consists of the designed MWC module, which integrates multiple wavelet kernels to extract specific fault-related information. This is followed by three ModernTCN blocks that comprehensively learn temporal relationships and high-dimensional features. Simultaneously, the original input signals are fed into the selective fusion module to generate adaptive weights, which determine the participation of different wavelet kernels based on the data distribution characteristics. The final output classifications are optimized against ground truth labels using a cross-entropy loss function, which effectively constrains the model to converge toward optimal diagnostic performance. This integrated architecture enables systematic feature learning from both frequency and temporal domains while maintaining adaptive kernel selection based on signal characteristics. The detailed structure parameters of proposed selective wavelet kernel fusion network are shown as Table 1.

## 3. Experimental Results

### 3.1. Data Description

**Figure 4:**
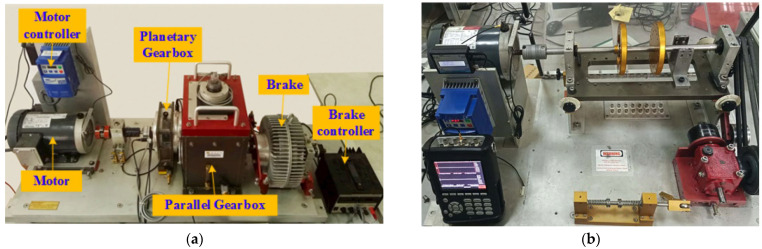
The detailed structure of test rig. (**a**) SEU dataset [18]. (**b**) SQ dataset [19].

In this paper, we utilize two open gearbox datasets to validate the performance of proposed selective kernel fusion neural network. The first dataset is a gearbox fault dataset provided by Southeast University (SEU dataset) [18]. The test rig consists of a three-phase induction motor with a 3 HP capacity serving as the drive source, a single-stage planetary gearbox, a parallel shaft gearbox, and a programmable magnetic brake. An accelerometer was mounted on the input side of the planetary gearbox to collect vertical vibration signals at a sampling frequency of 25.6 kHz. Vibration data were acquired under five distinct health conditions, with detailed descriptions provided in Table 2 and Table 3. The continuous signals were segmented using a non-overlapping sliding window approach, resulting in 1023 samples per condition and a total of 9207 samples. Each sample contains 1024 data points. The second dataset is a bearing fault dataset provided by Spectra Quest (SQ dataset) [19], which includes seven distinct health conditions with varying severity levels. Detailed descriptions of these conditions are summarized in Table 3. The vibration signals were acquired at a sampling frequency of 25.6 kHz. Through a segmentation process applied to the raw signals, a total of 5390 samples were obtained, with each health condition comprising 630 samples. In selecting the datasets, two types were chosen: the SEU dataset to evaluate recognition capabilities across different fault categories, and the SQ dataset to assess recognition capabilities across varying degrees of fault severity. Moreover, the focus of one dataset is gears, whilst the other concentrates on bearings, thus providing a comprehensive coverage of the fundamental structure of the gearbox, see Figure 4.

**Table 2:** The detailed fault type of SEU dataset.

Fault Type	Label
Normal	Normal
Chipped	Fault1
Miss	Fault2
Root	Fault3
Surface	Fault4

**Table 3:** The detailed fault type of SQ dataset.

Fault Type	Label
Inner race—light damage	IF1
Inner race—medium damage	IF2
Inner race—heavy damage	IF3
Outer race—light damage	OF1
Outer race—medium damage	OF2
Outer race—heavy damage	OF3
Normal	N

### 3.2. Experiment Setup

For both datasets used in the experiment, the samples were randomly split into training, validation, and test sets in a 6:2:2 ratio to ensure adequate training and reliable evaluation of the deep learning models. All models were trained and evaluated using the same hyperparameter settings, as detailed in Table 4, to guarantee a fair comparison. The models were evaluated using three standard performance metrics: accuracy, precision, and recall. The formulas for these metrics are shown as below:

(12) $$ACC=\frac{TN+TP}{TN+TP+FN+FP}$$

(13) $$Precision=\frac{TP}{TP+FP}$$

(14) $$Recall=\frac{TP}{TP+FN}$$

where TP, TN, FP and FN are the true positive, true negative, false positive and false negative, respectively.

All experiments were conducted on a computer equipped with an Apple M4 CPU and running the 64-bit macOS Tahoe operating system. The algorithms were implemented in Python 3.7 using the CPU edition PyTorch 1.14 library.

### 3.3. Performance Evaluation

This section employs an empirical approach to assess the validity of the proposed method by means of two experimental datasets. Specifically, the SEU dataset is utilized to assess gear diagnostic capability, while the SQ dataset is employed to evaluate bearing fault diagnosis capability. The method proposed in this paper constitutes an enhancement upon existing CNN-based architectures. To provide an adequate foundation for comparison, four CNN-type networks were selected: one baseline CNN and three modified CNNs. The CNN is regarded as the foundational architecture. MA1DCNN [20] constitutes a multi-layer stacked network that integrates temporal and spatial attention mechanisms. ResNet [21] serves as a quintessential and remarkably effective model in the domain of computer vision. ResNet-CBAM further refines the attention mechanism inherent to the ResNet foundation, thereby enhancing the model’s capacity to discern critical information within vibration signals.

The following Table 5 and Table 6 offers a synopsis of the mean classification accuracy results obtained by disparate methodologies across five replicate experiments. These experiments utilized various working conditions and focused on a particular dataset. The experimental findings demonstrated that across the five trials, the SWKFNN model consistently achieved a maximum classification accuracy that surpassed 99%, thereby showcasing a markedly superior performance in comparison to other convolutional neural network-based models (CNN, MA1DCNN, ResNet, ResNet-CBAM). Conventional CNN models demonstrate the least optimal performance due to their propensity to allocate equal attention to all regions and information present within the input, thereby neglecting to dynamically focus on key features. Consequently, the classification accuracy of these models remains suboptimal. Two enhanced CNN-based models exhibited superior performance in comparison to the baseline CNN architecture, achieved through structural optimizations: MA1DCNN incorporated an attention mechanism module, while ResNet introduced residual connections within its deep network architecture. The enhanced performance of these models, as evidenced by their superior performance in comparison to traditional CNN models, has been demonstrated by means of specialized structural optimization. The ResNet-CBAM model, by incorporating a convolutional block attention module into the ResNet architecture, enhances its ability to capture key features via an “attention mechanism,” achieving classification accuracy second only to the SWKFNN model.

**Figure 5:**
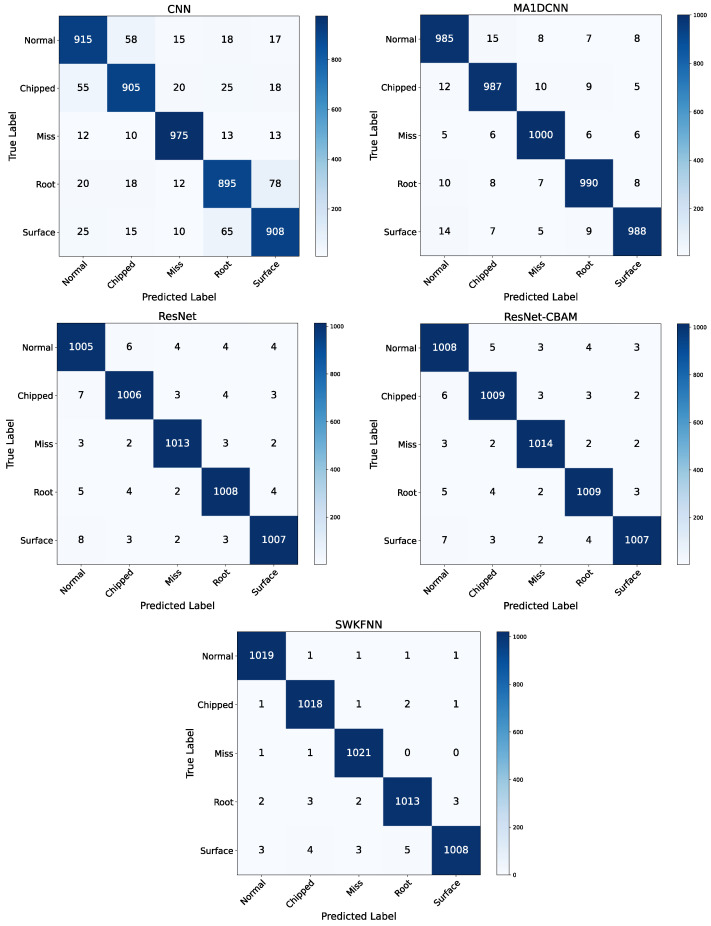
Confusion matrix comparison on SEU dataset.

Furthermore, the classification performance of different methods under various conditions was investigated by plotting the confusion matrix results for the SEU dataset during operation as shown in Figure 5. The SWKFNN model proposed in this study demonstrates a high degree of accuracy in identifying healthy conditions, with a success rate exceeding 99%. However, it is important to note that the system is not capable of fully diagnosing certain older faults, which can result in isolated misclassifications. As demonstrated in the case of traditional CNN models, there is room for improvement with regard to the performance of these models in diagnosing composite faults. The accuracy rates of 88.1% and 88.9% that are obtained for the third and fifth fault diagnoses, respectively, are indicative of this suboptimal performance. MA1DCNN, ResNet, and ResNet-CBAM show significant enhancement to the effectiveness of composite fault diagnosis due to their specialized structural designs, which are engineered to optimize the extraction and utilization of diagnostic information. Among these approaches, the MA1DCNN design is distinguished by its incorporation of an Excitation Attention Module (EAM). The present module has been designed to simulate the process of capturing excitation information within signals by way of detecting abrupt changes in the time domain. This module has been demonstrated to exhibit sensitivity to pulse signals and has achieved significant improvements for single fault categories. ResNet employs residual learning by integrating residual blocks. Convolutional layers, in conjunction with multiple residual blocks, comprise a residual connection design. This design facilitates depth augmentation through the use of stacked residual blocks without compromising the efficacy of fault diagnosis. ResNet-CBAM is an enhancement to ResNet that incorporates a Convolutional Block Attention Module (CBAM). The model exhibits a minimal computational overhead, which allows it to combine channel attention and spatial attention mechanisms. This combination enhances the model’s focus on effective features, thereby further improving classification accuracy. ResNet-CBAM exhibits a high degree of proficiency in fault diagnosis, attaining classification accuracy of nearly 99% for healthy states. The diagnostic performance of the model under consideration approaches that of the proposed SWKFNN model, though with a slightly increased computational load and inferior model stability. A comparison of traditional CNN models with other deep learning architectures reveals that the former exhibit significantly lower classification accuracy and stability. This finding underscores the merits of deep structures in machine learning. The proposed SWKFNN model exhibits superiority in accuracy and stability when compared with alternative approaches. This finding indicates that the integration of selective wavelet kernels with neural networks has yielded a stable and precise fault diagnosis model.

**Table 5:** Related Methods Comparison on SEU dataset.

Model	Mean ± Var	1	2	3	4	5
CNN	89.1112 ± 0.5235	89.1283	89.1398	88.1775	90.1982	88.9122
MA1DCNN	96.1997 ± 0.2872	96.3287	96.8734	96.1879	95.3768	96.2321
ResNet	98.4521 ± 0.1055	98.2876	98.1969	98.9122	98.1894	98.6746
ResNet-CBAM	98.7870 ± 0.1143	98.2319	99.1093	98.8965	98.9627	98.7349
SWKFNN	99.3746 ± 0.0766	99.1270	99.5691	99.2387	99.1767	99.7619

**Table 6:** Related Methods Comparison on SQ dataset.

Model	Mean ± Var	1	2	3	4	5
CNN	85.3931 ± 0.0724	85.7125	85.3791	85.6112	85.1709	85.0921
MA1DCNN	93.9385 ± 0.3332	93.2135	94.2087	93.4819	94.1593	94.6293
ResNet	97.3221 ± 0.0744	97.0904	97.6128	97.1279	97.1527	97.6271
ResNet-CBAM	97.7487 ± 0.1583	97.8731	97.0912	98.1723	97.7962	97.8108
SWKFNN	98.1354 ± 0.0225	98.0172	98.3891	98.1019	98.1371	98.032

### 3.4. Ablation Study

Convolutional neural networks (CNNs) are susceptible to limitations in their capacity to express features during the process of feature extraction. Consequently, the present study employed an ablation analysis to validate the hypothesis that the introduction of different kernel functions and kernel fusion strategies enhances CNN performance. This finding confirms the effectiveness of the proposed SWKFNN. The present study utilizes an ablation experiment to investigate the impact of distinct kernel functions on model performance. The integration of diverse kernel functions, including Sine, Mehta, and Laplace, along with kernel fusion through Wavelet, within the CNN framework, serves to substantiate the individual contributions of each constituent element to the overall model, designated as SWKFNN. The analysis is conducted across two datasets (SEU and SQ). The experiments compared the accuracy (ACC), precision, and recall of the following models: ‘only-CNN’, ‘single-kernel-CNN’, ‘kernel-fusion-CNN’, and ‘SWKFNN’.

As shown in Table 7, experiments on the SEU dataset demonstrate that the only-CNN model performed the worst, indicating that basic CNN models possess limited capability to capture features within the dataset. The introduction of three single-kernel Convolutional Neural Networks (CNNs) demonstrated that the SineKernel-CNN augmented periodic feature extraction relative to the CNN, thereby enhancing the accuracy and aligning more closely with the characteristics of vibration data. However, the fault dataset predominantly comprised fault data, manifesting diminished periodicity. Consequently, both the MexhetKernel-CNN and the LaplaceKernel models demonstrated superior performance in comparison with the SineKernel-CNN, with the LaplaceKernel-CNN exhibiting a particularly notable advantage. Laplace, a unilaterally right-decaying wavelet, exhibits a superior alignment with the incremental dissipation of anomalous influences, thereby demonstrating the most efficacious fault information extraction capability and enhancing the accuracy to exceed 98%. The Wavelet Kernel Fusion-CNN demonstrates superior accuracy when compared to the optimal single-kernel CNN (Laplace), thus substantiating the hypothesis that multi-kernel fusion complements features to further enhance model performance. The SWKFNN model that is the subject of this study has been demonstrated to be the most effective of all the models that have been improved. Building upon the foundation of kernel fusion, its comprehensive optimization design serves to further unlock the feature potential.

The performance trends observed in the SQ dataset are consistent with the trends seen in the SEU dataset, as shown in Table 8, although there are minor discrepancies in the absolute values. This discrepancy originates from the SQ dataset, which represents bearing fault severity. Early-stage faults and normal samples exhibit minimal distinguishability in this dataset, thereby posing a significant challenge to the model. It is noteworthy that Only-CNN remains the least effective model among the six optimised designs. In the context of single-kernel CNNs, the utilization of the Laplace kernel consistently yields optimal outcomes. The WaveletKernelFusion-CNN demonstrates superior performance in comparison to the three individual CNNs, while the proposed SWKFNN attains the optimal model optimization outcome for this particular dataset.

The present ablation study offers compelling evidence that the incorporation of kernel functions—particularly the Laplace kernel—significantly enhances the performance of CNNs. This finding establishes kernel functions as pivotal components for enriching feature representation. Multi-kernel fusion CNNs have been shown to outperform single-kernel CNNs, suggesting that features derived from multiple kernels exhibit complementary properties that can mitigate the limitations of single-kernel CNNs. The proposed SWKFNN represents a refinement of multi-kernel fusion, emerging as the optimal model with optimal performance. This outcome serves to validate the effectiveness of the proposed design.

### 3.5. Cluster Visualization Analysis

**Figure 6:**
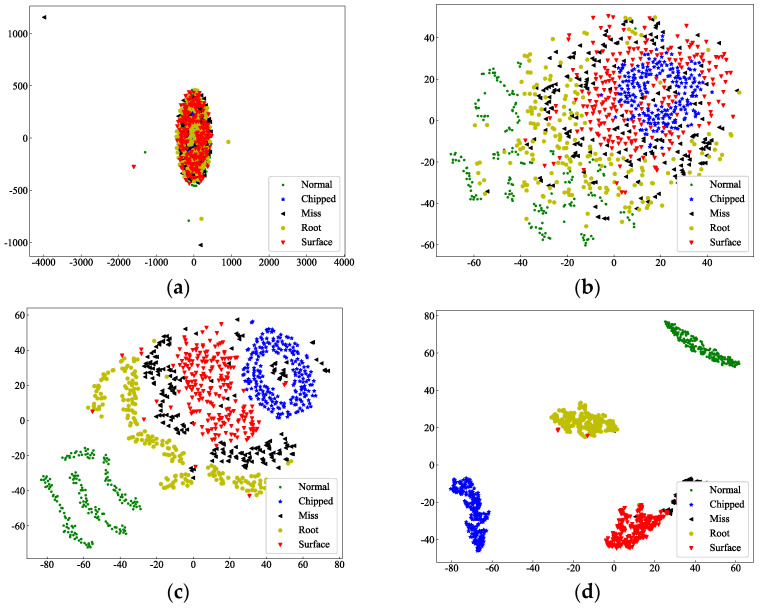
Cluster visualization of learning process. The following data is required for the purpose of visualizing the feature clustering using tSNE: (**a**) The raw data (**b**) The selective fusion feature (**c**) The TCN feature (**d**) The classification feature.

To conduct the feature learning ability and classification ability, we plot the tSNE figure for visualization of cluster process. As illustrated in Figure 6, the data samples progressively develop clustering capability within the feature space. Figure 6a displays the distribution of raw data samples, where all categories are completely intermingled without any discernible separability. This observation fundamentally underscores the necessity for diagnostic technology to advance from manual approaches toward intelligent automated systems. Figure 6b presents the feature distribution after selective fusion, where samples begin to exhibit preliminary separation trends layer by layer through the multi-wavelet convolutional kernel selective fusion. The emerging clustering patterns indicate that the model has already acquired considerable diagnostic capability at this stage. In Figure 6c, which shows the data distribution after the fused features undergo temporal relationship learning and high-dimensional feature extraction through standard convolutional layers, the boundaries between different categories become more distinct and the feature clusters grow increasingly compact. Figure 6d demonstrates the final output distribution of the model, where the data becomes completely separable, signifying a substantial enhancement in diagnostic performance. This feature clustering visualization experiment convincingly validates the learning capacity of the proposed method. The model progressively refines its understanding of the data and features at each layer, effectively learning critical fault-related information while continuously improving its diagnostic classification capability throughout the hierarchical learning process.

### 3.6. Parameter-Sensitive Analysis

**Figure 7:**
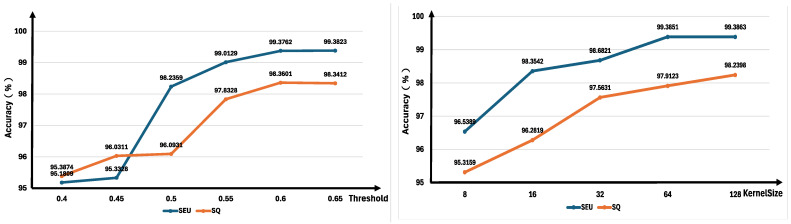
Parameter analysis results of threshold and kernel size.

The present study’s parameter-sensitivity analysis principally concentrates on two primary parameters. Two further variables must be considered: KernelSize and threshold. The analysis results are shown in Figure 7. The study meticulously examined the performance variations across the SEU and SQ datasets, thereby elucidating the parameters’ sensitivity to the model and ascertaining their optimal values.

KernelSize represents the fundamental parameter of the kernel function, directly impacting the granularity of feature extraction. The experimental results confirmed the validity of sensitivity analyses for KernelSize across five values: The values considered were 8, 16, 32, 64, and 128. The experimental results demonstrate that as KernelSize increases from 8 to 64, the model’s performance undergoes a substantial enhancement. However, within the range of 64 to 128, the observed performance enhancements either reach a plateau (SEU) or manifest only a modest improvement (SQ). The findings suggest that 64 is the optimal kernel size for the SEU dataset, attaining nearly peak performance with considerable cost-effectiveness. Conversely, 128 signifies the optimal kernel size for the SQ dataset, exhibiting marginal potential for further enhancement. It is imperative that the kernel size be aligned with the feature scale of the dataset. The SEU dataset’s effective features are fully captured at scale 64, and larger kernels yield no additional benefit. Conversely, the SQ dataset exhibits a richer feature scale, requiring scale 128 for comprehensive coverage.

The threshold is of critical importance in the context of model decision-making. The experimental procedure involved the assessment of six distinct values: the values considered were 0.4, 0.45, 0.5, 0.55, 0.6, and 0.65. Experiments demonstrated that between thresholds of 0.4 and 0.6, there was a concomitant improvement in model performance with increased threshold values. Nevertheless, beyond an optimal level of 0.6, performance exhibited a plateauing effect on the SEU dataset, accompanied by a slight decline on the SQ dataset. Consequently, 0.6 represents the optimal threshold for both SEU and SQ datasets, at which point SEU performance approaches its peak and SQ performance attains its maximum. These experiments demonstrate that thresholds that are excessively low can result in misclassifications, where non-targets are incorrectly identified as targets. Conversely, thresholds that are excessively high can lead to false negatives, wherein targets are incorrectly classified as non-targets. A threshold of 0.6 represents the optimal balance point between misclassification and false negatives. Beyond this threshold, the risk of false negatives increases.

### 3.7. Noise Robustness Analysis

**Figure 8:**
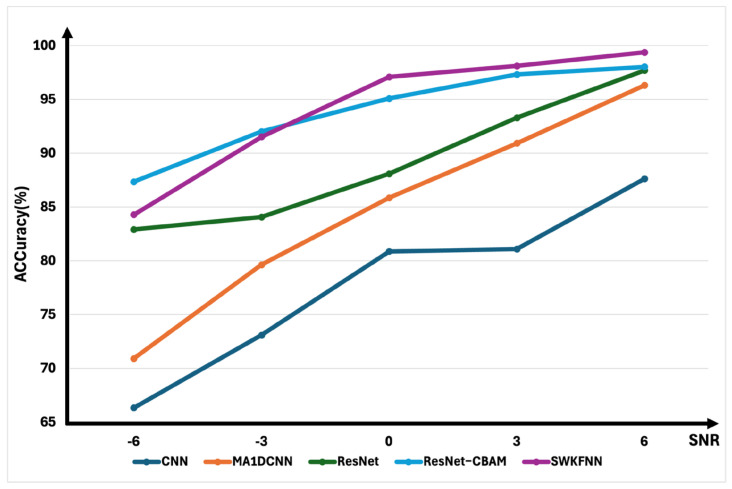
Noise robustness results under different SNR.

The present study sought to investigate the impact of signal-to-noise ratio (SNR) on the performance of five models in simulated environments characterized by varying levels of data interference. The experimental results are shown in Figure 8. The experiments were designed to emulate real-world conditions by introducing noise into the data, with higher SNR values signifying more pronounced noise interference. The objective was to assess the resilience of the models to such perturbations and to determine the extent to which they can withstand interference in noisy settings. The core metric of this study was model accuracy, which is defined as follows: higher values denote greater robustness. The experimental results demonstrated that baseline models, such as CNN and MA1DCNN, exhibited extreme sensitivity to noise, with performance exhibiting significant fluctuations as SNR increased. In contrast, deep models, such as ResNet and ResNet-CBAM, demonstrated a marked superiority in noise resistance. The convolutional block attention mechanism of ResNet-CBAM further augmented interference resilience. The proposed SWKFNN model exhibited optimal performance across the entire noise range, thereby establishing itself as the most robust and noise-resistant model. This integration of wavelet kernels equips the model with the inherent capability to accurately extract relevant information under complex noisy conditions, representing a significant advantage of incorporating prior domain knowledge.

Experimental results under noisy conditions reveal a consistent trend: model performance improves with increasing signal-to-noise ratio (SNR), aligning with fundamental expectations. Traditional CNN models exhibit the lowest noise robustness across all SNR levels, while MA1DCNN shows moderate improvement yet remains susceptible to severe noise interference. ResNet architectures demonstrate significantly enhanced robustness owing to their deep structure, with ResNet-CBAM further strengthening noise resistance through attention mechanisms.

Under conditions of high-noise interference (SNR = −6), conventional CNN models demonstrate the poorest performance, exhibiting extreme sensitivity to substantial noise interference. MA1DCNN demonstrates a notable enhancement in performance compared to conventional CNNs; however, it exhibits a substantial vulnerability to severe noise interference. ResNet models exhibit significantly enhanced robustness, with their intricate deep architecture beginning to demonstrate resilience. The incorporation of the CBAM within ResNet-CBAM models has been demonstrated to further enhance the models’ resilience to noise, thereby leading to superior performance when compared to the base ResNet model. Notably, the proposed SWKFNN, despite having only five layers, achieves performance comparable to the much deeper ResNet models and substantially outperforms traditional CNNs. This indicates that the integration of wavelet kernels and selective fusion mechanism effectively compensates for the model’s structural simplicity in challenging noise environments. As noise decreases, the proposed method gradually demonstrates superior performance, ultimately achieving the highest diagnostic accuracy under mild and minimal noise conditions.

## 4. Conclusions

This paper has proposed a knowledge-guided selective wavelet kernel fusion neural network to address the limitations of conventional deep learning methods in gearbox fault diagnosis. By integrating domain knowledge of wavelet analysis with data-driven modeling, the developed approach enhances both interpretability and diagnostic performance while overcoming the black-box nature of traditional deep learning models. The main accomplishments of this work can be summarized as follows: First, the multi-wavelet kernel convolution module provides prior interpretability through physically meaningful feature extraction, directly addressing the interpretability challenge in deep learning-based diagnosis. Second, the integration of MWC with ModernTCN enables comprehensive temporal feature learning while maintaining the benefits of wavelet analysis. Third, the attention-based selective fusion mechanism adaptively optimizes kernel selection according to data distribution characteristics, ensuring optimal feature extraction for different fault conditions. Experimental validation on two public gearbox datasets demonstrates that the proposed method not only achieves superior diagnostic accuracy compared to conventional approaches but also provides transparent decision-making process through its knowledge-guided architecture.

Future work will focus on extending the proposed framework to other critical components in industrial systems and exploring its application under more challenging operational conditions, including variable speeds and loads. Additionally, we plan to investigate the integration of more diverse domain knowledge sources to further enhance the diagnostic capability and interpretability of the system. Concurrently, the challenge of collecting comprehensive fault samples within industrial settings is a significant impediment, as it precludes the possibility of obtaining samples for all fault categories. This, in turn, leads to a deficiency in the identification capability for unknown faults. This approach shows great potential for future expansion into the domains of zero-shot learning and incremental learning, enabling the acquisition and recognition of novel fault categories.

## Figures and Tables

**Table 1:** Structure parameters of selective wavelet kernel fusion network.

Module	Parameter	Value
MWC	Kernel size	64
	Output channel	32
ModernTCN	Input channel	32, 64, 64
	Dilated ratio	3, 5, 7
	Output channel	64, 64, 128
	Kernel size	63, 63, 32
Fully connected	Node	128, 64, class number

**Table 4:** Parameter setting.

Parameter	Value
Experiment repeat	5
Batchsize	64
Learning rate	0.005
Epoch	150
Optimizer	NAdam

**Table 7:** Ablation study results on SEU dataset.

Model	Only-CNN	SineKernel -CNN	MexhatKernel -CNN	LaplaceKernel -CNN	WaveletKernel Fusion-CNN	SWKFNN
ACC	89.1876	92.3462	93.6582	98.1976	98.5341	99.3786
Precision	89.3287	92.3473	93.6589	98.1970	98.5322	99.3791
Recall	89.1876	92.3462	93.6582	98.1976	98.5341	99.3786

**Table 8:** Ablation study results on SQ dataset.

Model	Only-CNN	SineKernel -CNN	MexhatKernel -CNN	LaplaceKernel -CNN	WaveletKernel Fusion-CNN	SWKFNN
ACC	83.1709	88.6523	90.0871	97.2861	98.0716	98.3967
Precision	83.1728	88.6541	90.0882	97.2865	98.0793	98.3984
Recall	83.1709	88.6523	90.0871	97.2861	98.0716	98.3967

## Data Availability

The authors confirm that the data underlying the results presented in this study, are available from the corresponding author upon reasonable request.
